# Prehospital Use of Ultrasound by Paramedics: A Literature Review

**DOI:** 10.7759/cureus.77388

**Published:** 2025-01-13

**Authors:** Melissa Mallary, Janelle Trefsgar, Meriah Parker, Isabella Hobbs, Avery Love, Inder Makin

**Affiliations:** 1 Emergency Medicine, A.T. Still University - School of Osteopathic Medicine in Arizona, Mesa, USA; 2 Obstetrics and Gynecology, A.T. Still University - School of Osteopathic Medicine in Arizona, Mesa, USA; 3 Pediatrics, A.T. Still University - School of Osteopathic Medicine in Arizona, Mesa, USA; 4 Internal Medicine, A.T. Still University - School of Osteopathic Medicine in Arizona, Mesa, USA; 5 Research, A.T. Still University - School of Osteopathic Medicine in Arizona, Mesa, USA

**Keywords:** diagnostic ultrasound, ems, paramedics, pocus, prehospital, ultrasound review

## Abstract

Ultrasound technology is widely used in hospital settings throughout the United States to aid in the diagnosis and treatment of various diseases. While the benefits of utilizing ultrasound in hospital settings are well understood, there are prehospital scenarios where the utility of ultrasound could be examined. Prehospital care often addresses acute and time-sensitive medical conditions, in which additional diagnostic information and early pathology detection can be crucial. Given the nature of these conditions, the use of ultrasound by paramedics in the prehospital setting is considered. This narrative review assesses the benefits and challenges of implementing widespread prehospital ultrasound use.

## Introduction and background

Role of imaging

Healthcare professionals (HCWs) are tasked with correctly diagnosing and treating patients based on their presenting symptoms. However, the clinical presentation may not fully explain the disease process that the patient is experiencing. HCWs must utilize other diagnostic modalities, such as imaging, to better understand the underlying cause of a patient's illness. Medical imaging allows for visualizing internal tissues and organs to investigate potential pathologic anatomy and physiology that could explain the patient's clinical presentation [1].

Various imaging modalities find application in healthcare, including X-ray radiography, X-ray computed tomography (CT), magnetic resonance imaging (MRI), ultrasound (US) imaging, and several others [1]. Nonetheless, most of these healthcare imaging modalities are characterized by their substantial size, requirements for restricted patient movement, and lack of portability [1]. Lack of portability poses constraints on the accessibility of these technologies, especially in urgent prehospital scenarios [1]. US stands out as the imaging modality with the greatest portability [1]. However, diagnostic US is operator-dependent and requires proper training. Furthermore, in many situations, US is a rule-in tool rather than a rule-out tool, like in the case of hemoperitoneum [1].

US history and physical basics

US has been used in medicine since the mid-20th century [2]. It is a derivative of previously established sound navigation and ranging (SONAR) technology from submarine vessels, combined with the emerging advancements in computer chips and advanced signal processing capabilities [2]. Since its introduction, US has become an increasingly relevant imaging modality. This is partially due to mitigating the risk of ionizing radiation from other imaging technologies, improving US machinery's portability, and lowering costs [2].

US technology uses sound waves ranging between 2 and 15 MHz, also referred to as ultrasonic waves, that are inaudible to the human ear [2]. As US waves pass through tissue, a property called acoustic impedance, which is specific to each medium, determines the amount of energy that passes through or is reflected to the transducer. Attenuation of the US waves will occur based on how the waves interact with the varying media due to absorption, scattering, reflection, and refraction [2]. By projecting US waves at/through objects of various densities and compositions, the signal is reflected to the transducer differently [2]. These returned signals are then interpreted with computerized assistance to produce images that reflect the physical properties of the biological matter the signal passes through [2]. 

Tissue boundaries that reflect most of the incident acoustic energy, such as bone, appear hyperechoic (bright/white) on imaging [2]. Materials or structures that are highly attenuative reflect a weak echo signal, represented as hypoechoic, appearing dark/black on imaging. By understanding the principles of echogenicity as it applies to various anatomical structures, US imaging can be utilized to visualize underlying structures for diagnostic and procedural purposes [2]. These principles of echogenicity can be applied in identifying unique signatures of pathology, differentiating it from normal tissue [2].

Evolution of technology and adoption

Technological advances in US imaging have enabled the development of portable imaging systems suitable for prehospital care, which includes the emergency medical services (EMS) provided to patients before their arrival at a hospital [3]. In the United States, prehospital care is traditionally provided through EMS personnel, with paramedics providing the highest level of care [4]. Paramedics are trained in basic and advanced skills that are focused on the acute management of varying patient presentations [4]. Their scope of practice includes medical interventions such as airway and breathing management, pharmacological treatments like establishing intravenous access and administering blood transfusions, and cardiac care [4]. In addition, paramedics are tasked with transporting patients to appropriate medical facilities for treatment based on predominant patient disposition [4].

This paper aims to review previous research on the use of US in prehospital settings, specifically exploring the limitations of integrating point-of-care ultrasound (POCUS) into these environments. The objective is to determine whether POCUS should be incorporated into the EMS scope of practice. This review expands upon prior studies that were more narrowly focused on the use of prehospital ultrasound in trauma situations or its application in specific settings such as air medicine, ground advanced life support, remote EMS, and military scenarios [5,6].

## Review

Advantages

The analysis of US utilization by EMS in previous studies highlights the notable advantages of this diagnostic tool, especially in accelerating the detection and handling of life-threatening conditions, such as hemoperitoneum and tension pneumothorax, in prehospital scenarios [5]. Between 2008 and 2009, a research initiative was undertaken by trained paramedics to assess the advantages and precision of US utilization [7]. The investigation concentrated on employing Focused Assessment Sonography for Trauma (FAST) and abdominal aortic (AA) examinations [7]. Conducted in two Minnesota cities, the study involved 104 patients, encompassing 84 FAST exams and 20 AA exams [7]. Trained paramedics conducted prehospital US assessments on indicated patients, and positive findings were corroborated by operative or CT results. Results revealed that all positive prehospital US were concordant with confirmed evaluations [7]. The investigators acknowledged study limitations, such as non-randomized sampling and non-blinded paramedics, potentially introducing bias [7]. Nonetheless, it concluded that prehospital US by trained paramedics could enhance patient outcomes by expediting the diagnosis of potentially life-threatening conditions [7].

Other prior studies on US in prehospital settings focus on paramedics using and interpreting US to hasten the diagnosis of life-threatening conditions in trauma scenarios, such as hemoperitoneum and tension pneumothorax [5]. A study out of Pittsburgh, Pennsylvania, involving two ambulance stations, explored the feasibility of paramedic-performed lung US for patients with respiratory distress and hypoxia [8]. Paramedics were tasked to obtain US images, which were then interpreted remotely by an EMS physician [8]. The study, overall, determined that paramedics who obtained US images with EMS physician interpretation did not meet the feasibility criteria for real-world applications [8].

A case study in the American Journal of Emergency Medicine highlighted the utility of prehospital US by detailing how EMS utilized a portable US device to evaluate patients with post-jackhammer chest pain [9]. The use of US ruled out tension pneumothorax and diaphragm rupture, leading to the diagnosis of atelectasis [9]. The authors emphasized the diagnostic and therapeutic advantages of US in acute blunt thoracic trauma, which allows for the improved management and treatment of potentially lethal thoracic injuries [9].

The usefulness of portable US in multiple scenarios has led to several departments expanding their scope of practice to include POCUS. The standard for POCUS education for paramedics varies widely and does not align with qualification or level of clinical experience. Paramedics' qualifications range from short vocational courses to undergraduate and postgraduate degrees in developed countries [10]. POCUS has started to gain popularity in the United States [11]. However, there has been apprehension based on the cost of training and equipment and inconsistent evidence of improved outcomes with standard US usage [11].

Challenges with standardized training and deployment

Currently, US is not part of the standard paramedic curriculum in the United States [12]. To begin training as a paramedic in the United States, an individual must have an Emergency Medical Technician (EMT)-Basic certification and complete a course in anatomy and physiology. Further, the paramedic curriculum ranges from 1,200 to 1,800 hours [12]. It consists of four phases: Didactic instruction to cover cognitive material, a Skills Laboratory to develop psychomotor skills, Clinical Education to integrate both the cognitive and psychomotor skills in a clinical setting, and a Field Internship to evaluate these skills under close supervision of an evaluator [12]. Lastly, there is a national exam to pass to obtain licensure from the State Office of Emergency Medical Services [12]. The paramedic curriculum builds upon EMT education to advance their skills. Paramedic students learn to administer medications, start intravenous lines, manage advanced airways, interpret electrocardiograms, and navigate life-threatening emergencies [13].

A 2014 survey in the United States found that only 4.1% of EMS departments currently utilize US, with 21.7% of departments considering its implementation [14]. The continued reduction in the size of standard US machines has led to the development of handheld, portable US devices. The first portable US device was available in 1975, followed by the first battery-powered pocket-sized US device in the late 1990s [15]. The most recent advances in handheld US technology have been the production of smaller and lighter devices with higher-quality imaging [15]. As the popularity of US use by EMS rises and the curriculum for its use is further refined, there may be a rise in departments that adopt its use. Potentially, recognition of the clinical utility of POCUS at the national level through the National Registry of Emergency Medical Technicians (NREMT) will further enhance its role in prehospital deployment [14,15].

In a scoping review by Meadley et al., 18 articles were analyzed, 13 of which were in the United States, to describe the training in POCUS for paramedics outside of the hospital setting [10]. Educational courses ranged anywhere from minutes to days to weeks. The settings varied from online courses to in-person, hands-on sessions. All training included didactic and practical sessions. POCUS simulations involved healthy volunteers, swine specimens, artificial models, and/or cadavers [10]. Assessments were conducted through written examinations, image interpretation, and objective structured clinical examinations (OSCEs) by bedside US-trained emergency physicians in the emergency department [8,16]. Competency and a minimum number of scans are used as an educational metric in other healthcare professions. The American Institute of Ultrasound in Medicine recommends 50 procedures annually and continuing medical education (CME) for physicians to maintain US skills [17,18]. For example, physicians who utilize POCUS require 10 CME credits every three years and 50 cases every 12 months [17,18]. The American Academy of Family Physicians recommends 150-300 reviewed scans for general competency, 25-50 scans for any specific examination, and 5-10 scans for US procedural guidance based on emergency medicine requirements [17]. Meanwhile, the American College of Emergency Physicians (ACEP) recommends 10 hours of CME every two years for emergency physicians [18]. However, this may not be generalizable to the prehospital arena due to the differences in initial education, the extent of US knowledge base, and the frequency of use [18].

Various studies to date have detailed the different ways to teach US and test participant competency [16,19-21]. A pilot study by Rooney et al. revealed that a three-hour training program for paramedics, comprising two hours of lessons and one hour of hands-on practice, enabled them to produce clinically useful images in 89% of cardiac arrest cases by accurately distinguishing between cardiac activity and standstill, providing critical guidance for resuscitation efforts [19]. Another study by Chin et al. involved teaching 20 emergency-trained paramedics to perform and interpret US imaging of life-threatening conditions such as pneumothorax, pericardial effusion, and cardiac standstill [16]. A two-hour US training session was provided using the novel Prehospital Assessment with UltraSound for Emergencies (PAUSE) protocol, which involves a pleural line exam and a focused transthoracic echocardiogram [16]. The researchers concluded that the paramedics could adequately perform and recognize the presence of life-threatening conditions [16]. In teaching the FAST exam, Paddock et al. found no significant differences in image acquisition in patients among US users who were trained through traditional in-person didactics, remote simulation training, or a mixture of the two, suggesting that US skills can be taught through different instructional modalities [20]. Lastly, the results of a longer-established curriculum focused on extended FAST exams were examined by Press et al. [21]. In this study, the paramedics underwent a two-phase training involving a one-day course of two-hour didactics and four hours of hands-on training, followed by supervised and unsupervised scanning on patients and 60-120-minute online lectures [21]. At the end of the study, 28 of 33 paramedics were able to pass their post-test and OSCE, proving that the implementation of FAST exams in prehospital training is achievable [21].

While the goal of prehospital POCUS is to aid in the diagnosis and treatment of patients with no familiarity with POCUS among these personnel, a significant concern is the potential for delay in care. For instance, there is no standard US curriculum for paramedics, which limits US reliability and standardized use in the prehospital setting. There is also limited data on the effect of incorrect diagnoses from prehospital US findings. Therefore, negative findings should be further ruled as not being "false negative," which may impede resuscitation and other interventions [17,22]. This is specific to POCUS performed on-scene rather than in transit. It appears that using POCUS in conjunction with other procedures during transport did not pose a significant delay [22]. Yet there are still cases where the patient's condition is deemed too acute to complete a US exam [8].

The environment in which the US machine is being used influences the feasibility of prehospital POCUS. The machine is yet another item that needs to be on hand and transported if used on-scene. The size of the machine may present a challenge when used in confined spaces and when limited to one-handed operation [17,22]. Extreme temperatures and weather, such as precipitation, must also be considered, as paramedic work is not subjected to only indoor conditions [22].

Finally, transport time must be considered. Whether it be transportation by air or ground, the distance traveled is subjective to each case. Although a shorter transit time to the nearest hospital is beneficial for patient care, it may be too short to perform POCUS. However, this typically pertains to urban settings with shorter transport times, and it can be presumed that rural EMS may have more time to perform POCUS [6,22]. Nonetheless, delays in patient transport are non-existent when POCUS is employed in parallel with other necessary procedures [22].

## Conclusions

Integration of US imaging in prehospital settings, particularly within EMS, shows significant potential for the early detection and management of life-threatening conditions as evidenced by the positive outcomes reported in prior studies. However, the implementation of prehospital US faces notable challenges. The absence of a standardized US curriculum for paramedics in the United States, combined with varying levels of education and experience among practitioners, poses a barrier to widespread adoption. Concerns about consistent training, competency assessments, and ongoing education, aligning with other health professions, raise questions about the readiness of EMS personnel for effective US use. Additionally, logistical challenges related to using US machines in the prehospital environment, such as size constraints, potential delays in care, and environmental factors like extreme weather, need careful consideration. Balancing the need for rapid on-scene US exams with the imperative to prioritize life-critical patient care presents a complex challenge. The potential for false negatives and their impact on resuscitation efforts underscore the importance of further research and standardization of training and implemented protocols in this evolving field.

Despite these challenges, the ongoing advancements in US technology, particularly the development of portable and handheld devices, offer potential solutions to some logistical issues. The gradual expansion of various departments' scope of practice to include POCUS may lead to increased adoption, especially with refined curricula and evidence supporting improved patient outcomes. In conclusion, while prehospital US holds considerable promise, addressing current limitations, refining education and training protocols, and conducting further research are pivotal for its effective integration into prehospital care. As technology progresses and educational standards align, prehospital US has the potential to become a valuable asset in EMS, contributing to enhanced patient care and improved outcomes.
